# Supplementary figures and images for: CRISPR mediated transactivation in the human disease vector Aedes aegypti
Source: PLoS Pathog. 2023 Jan 19;19(1):e1010842. doi: 10.1371/journal.ppat.1010842 (PMC9888728; doi:10.1371/journal.ppat.1010842)

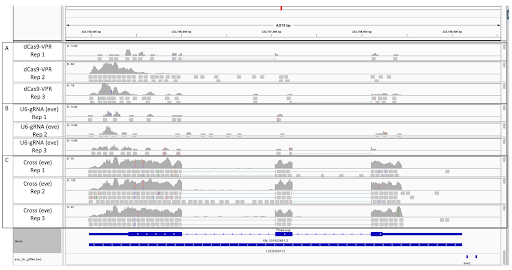

Supplement: S1 Fig — (PNG) [file ppat.1010842.s011.png]

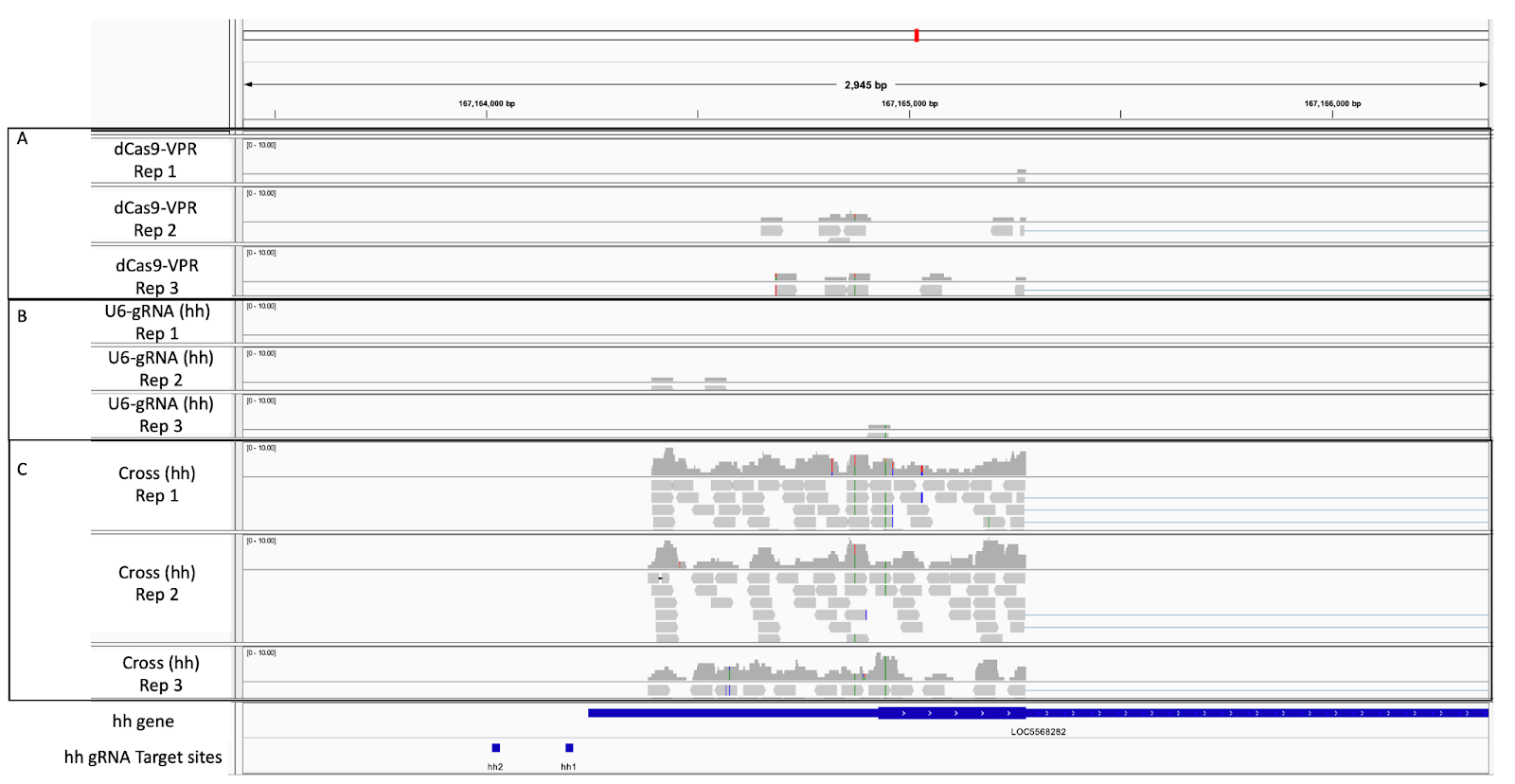

Supplement: S2 Fig — (PNG) [file ppat.1010842.s012.png]

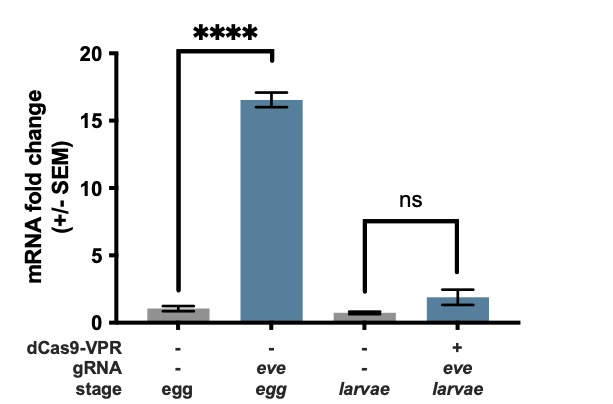

Supplement: S3 Fig — (TIFF) [file ppat.1010842.s013.tiff]
