# Supplementary material for: CRISPR mediated transactivation in the human disease vector Aedes aegypti
Source: PLoS Pathog. 2023 Jan 19;19(1):e1010842. doi: 10.1371/journal.ppat.1010842 (PMC9888728; doi:10.1371/journal.ppat.1010842)
Supplement: Attachment — Submitted filename: Review.docx [file ppat.1010842.s014.docx]

**Review for: “CRISPR Mediated Transactivation in the human disease vector *Aedes aegypti*”**

**Authors:** Bui M, Benetta ED, Dong Y, Zhao Y, Yang T, Li M, Antoshechkin IA, Buchman A, Bottino-Rojas V, James AA, Perry MW, Dimopoulos G, Akbari OS

**Reviewed by:** Michael Smanski, smanski@umn.edu

**Summary:**

The authors demonstrate CRISPRa-based activation of the *hh*, *eve*, and *rel1* genes in the disease vector *Aedes aegypti.* They describe the design and transgenesis of separate dCas9-VPR and sgRNA cassettes to make stable transgenic lines, the crossing of those lines to bring together dCas9-VPR and the sgRNAs in a single transheterozygote, and characterization of overexpression lines by RT-qPCR, RNAseq, Hybridization Chain Reaction in situ hybridization, and phenotypic assays.

This is a complete paper that provides compelling evidence that dCas9 activators can be used for biotechnological applications in *Ae. aegypti.* This is an impactful area of research with many possible applications stemming from this proof-of-concept. The manuscript is well-written and the figures are clear. The conclusions are well-supported by the data. Minor weakness, noted below, can be addressed during revision.

Overall, I recommend this paper for publication with minor revisions noted below.

Best,

Michael Smanski

smanski@umn.edu

**Weaknesses**

1. The most salient weakness is the lack of a control experiment to tease apart direct/indirect changes to the transcriptome revealed during RNAseq. Other groups that have investigated off-target gene activation with CRISPRa show very little off-target effects. As such, it is reasonable conclude that most of the differences in gene expression are indirect effects of the lethal- or near-lethal overexpression of hh/eve. Nonetheless there are several control experiments that could have been performed to provide direct evidence to support this.
2. Minor: With the RNAseq data in hand, the authors should discuss how rpl32 expression changes between samples (as this is used as a normalization reference gene in the RT-qPCR).
3. Minor: The authors should state how the heterozygous lines were created/identified prior to the matings that brought system components together. Specifically, the ‘Generation of transgenic lines’ section in the methods states that lines were maintained as mixtures of homozygotes and heterozygotes, where these outcrossed to WT to generate the parental heterozygous lines used, for example, in Figure 1B? Or were homozygotes separated out via some other means?
4. Figure 1, 4: numbers should be added to the DNA sequence to denote the positions of highlighted sequences relative to the transcription start site.
5. Figure 4E. It is unclear why median values are compared here instead of mean values
6. The references are a bit of a mess. Multiple papers are listed more than once, with the author’s 2021 Nat Comm paper listed at least 6 times.

**Minor revisions**

1. PG11; middle: H2O is written with a zero instead of the letter O.
2. Figure 3C: On a printed copy of the manuscript, data being shown in Figure 3c is undiscernible (and other fluorescent images are not much better). It looks better on a computer screen. Not sure if this can be fixed.
