# Supplementary material for: CRISPR mediated transactivation in the human disease vector Aedes aegypti
Source: PLoS Pathog. 2023 Jan 19;19(1):e1010842. doi: 10.1371/journal.ppat.1010842 (PMC9888728; doi:10.1371/journal.ppat.1010842)
Supplement: Attachment — Submitted filename: Responses to Reviewers_Letter.docx [file ppat.1010842.s016.docx]

December 12th, 2022

Dear Elizabeth A. McGraw, PhD and the PLOS Pathogens Editorial Team,

Thank you so much for providing us with the opportunity to revise our manuscript entitled “*CRISPR Mediated Transactivation in the Human Disease Vector Aedes aegypti*” for consideration as an article in PLOS Pathogens. We greatly appreciate the reviewers constructive comments, as we believe they have significantly improved our manuscript. We have addressed each of their points below (reviewer comments are in black text, our responses are in blue text, and any significant textual changes are pasted below for clarity).

Thank you for your consideration, and please let us know if we can provide any other information.

With best wishes,

Omar S. Akbari, Ph.D., Professor, Division of Biological Sciences, Department of Cell and Developmental Biology, University of California, San Diego, La Jolla, California 5101 Tata Hall, La Jolla CA, 92093, (858) 246-0640, oakbari@ucsd.edu

************************************************************************************************

Part I - Summary

Reviewer 1:

The authors demonstrate CRISPRa-based activation of the *hh*, *eve*, and *rel1* genes in the disease vector *Aedes aegypti.* They describe the design and transgenesis of separate dCas9-VPR and sgRNA cassettes to make stable transgenic lines, the crossing of those lines to bring together dCas9-VPR and the sgRNAs in a single transheterozygote, and characterization of overexpression lines by RT-qPCR, RNAseq, Hybridization Chain Reaction in situ hybridization, and phenotypic assays.

This is a complete paper that provides compelling evidence that dCas9 activators can be used for biotechnological applications in *Ae. aegypti.* This is an impactful area of research with many possible applications stemming from this proof-of-concept. The manuscript is well-written and the figures are clear. The conclusions are well-supported by the data. Minor weakness, noted below, can be addressed during revision.

Overall, I recommend this paper for publication with minor revisions noted below.

Best,

Michael Smanski

smanski@umn.edu

Dear Micheal,

We thank you for your comments and suggestions for improving our manuscript. We have addressed each of your points below:

1) The most salient weakness is the lack of a control experiment to tease apart direct/indirect changes to the transcriptome revealed during RNAseq. Other groups that have investigated off-target gene activation with CRISPRa show very little off-target effects. As such, it is reasonable to conclude that most of the differences in gene expression are indirect effects of the lethal- or near-lethal overexpression of hh/eve. Nonetheless there are several control experiments that could have been performed to provide direct evidence to support this.

The study has three controls represented by the parental lines: dCas and sgRNA lines etc. In total we sequenced 15 RNAseq samples which consisted of 3 replicates of the dCas9-Control; 3 replicates of the sgRNA eve ; 3 replicates of the sgRNA hh ; 3 replicates of the dCas9/sgRNA eve transhets ; and 3 replicates of the dCas9/sgRNA hh transhets (Table S2). By doing this many replicates we were able to conclude that the off target rate is quite low. We went back to the off-target analysis and saw that the 8 genes that look overexpressed in eve-transactivated embryos are also developmental genes or homeobox proteins indicating that likely the change in expression is due eve overexpression rather than off-targeting. This information is now included in the results and the section has been rephrased accordingly to improve clarity.

2) Minor: With the RNAseq data in hand, the authors should discuss how rpl32 expression changes between samples (as this is used as a normalization reference gene in the RT-qPCR).

Looking in Tables S1 to S6 the rpl32 gene expression (LOCLOC5577996) looks slightly downregulated in *hh* transhets (2.6 fold) indicating that the level of transactivation of *hh* is likely underestimated and that in reality it would be at much higher level. RNA sequencing is more accurate then comparing fold increase in hh transheterozygotes, the slight decrease in rpl32 explains the differences between TPM fold change and qPCR fold change (11 vs 8 fold respectively) for hh. No fold changes were detected in *eve* transheterozygotes.

3) Minor: The authors should state how the heterozygous lines were created/identified prior to the matings that brought system components together. Specifically, the ‘Generation of transgenic lines’ section in the methods states that lines were maintained as mixtures of homozygotes and heterozygotes, where these outcrossed to WT to generate the parental heterozygous lines used, for example, in Figure 1B? Or were homozygotes separated out via some other means?

We clarified how we performed our crosses in the Methods section: “To create true parental heterozygotes setting up transheterozygote crosses, PUb-dCas9 and sgRNA females were crossed to WT males. Resulting progeny positive for their respective fluorescent markers were collected and considered true transgenic heterozygotes. PUb-dCas9 females were crossed with sgRNA males. The reciprocal cross was also performed.”

4) Figure 1, 4: numbers should be added to the DNA sequence to denote the positions of highlighted sequences relative to the transcription start site.

Thank you for this suggestion. We have added numbers to the sequences shown in Figures 1 & 4 to denote their positions relative to the TSS.

5) Figure 4E. It is unclear why median values are compared here instead of mean values

The distribution of the viral titers is non-normal. We have had the same doubts in the past as the reviewer described above, and we have consulted JHSPH Biostatistical Department experts. Because of outliers, the median is used to describe a central tendency in non-normal data. A single extreme outlier can significantly skew the mean but will have little effect on the median. Therefore we have compared median values as our traditional statistical analysis standards, SEM was not included because mean values were not compared here. Non-parametric tests such as the Mann-Whitney test were used to effectively calculate the p-values and determine the statistical significance.

6) The references are a bit of a mess. Multiple papers are listed more than once, with the author’s 2021 Nat Comm paper listed at least 6 times.

References fixed

7) PG11; middle: H2O is written with a zero instead of the letter O.

We have corrected the typo.

8) Figure 3C: On a printed copy of the manuscript, data being shown in Figure 3c is undiscernible (and other fluorescent images are not much better). It looks better on a computer screen. Not sure if this can be fixed.

We have increased the brightness and contrast of the images seen in Figure 3C.

Reviewer 2:

This report describes the use of the CRISPRa system ( a 'dead' but guidable version of CRISPR-Cas9 fused to transcriptional activator) as a way to activate the expression of endogenous genes in the mosquito Aedes aegypti.

CRISPRa has been well used in many other systems, including the model insect Drosophila, but this is the first report of its use in a disease vector. Experiments are performed technically well, and I'm happy to accept that there is specific upregulation of genes targeted by CRISPRa.

1) The case for how this is enabling for future vector control applications however, could be better made. As could the case for how it is a step change over existing transactivation of transgenes through Gal4 or tTA etc, which are not really mentioned. Yes this is upregulating an endogenous gene, but tell us why this matters.

We thank the reviewer for this suggestion, we have rephrased part of the introduction and discussion to reflect his suggestion. In Particular we believe that transactivating genes in their endogenous locus will benefit the understanding of mosquito biology. It will be very useful for evaluating genes that have detrimental effects on development, reproduction, nutritional and metabolic processes, and other aspects of mosquito biology. Based on that information, vector control strategies can be developed. In addition the tools can be used to create synthetic species by creating stable reproductive barriers that produce an artificial selection pressure and drive genes through a population in a reversible manner. Based on this we have rephrased several parts of our manuscript.

1) In terms of the effects of CRISPRa activation, there are many global transcriptome changes, as would be expected from the misexpression of important developmental transcription factors but I'm not sure how much insight is added by these analyses. By upregulating an immunity transcription there is a real, but modest, effect on Dengue titre and prevalence. Again, the case for where this will lead in any application could be made more strongly.

The reviewer is right, overexpressing a developmental gene is causing major transcriptomic changes. Since it is a tool development paper, showing the wide effect that overexpressing the gene with this tool, represents a proof that gene function can be studied via transactivating gene expression using dCas9-VPR.

Reviewer 3:

In this manuscript, Bui et al describe their work on the development in Aedes aegypti, an important vector of arboviral diseases, of the CRISPa transactivation system for the overexpression/misexpression of endogenous genes. This system has previously been built in Drosophila and has been used both for basic research and for applications of vector control, as mentioned in this manuscript. The Akbari lab is a leader in the engineering of this mosquito, and other insects and the works build nicely on the previous work of this team. The authors clearly demonstrate that the system works as expected, based on the detection of target gene misexpression and, depending on the target gene, downstream misexpression of downstream targets affecting development and immunity.

1) In terms of novelty, this system was not previously available in this important insect, and now it is - whether that is sufficient novelty is questionable to this reviewer - but then novelty is partially subjective and so is usefulness. It is clear that the authors intended this mainly as a tool development paper demonstrating proof-of-concept, which they have succeeded in doing.

We agree with this reviewer that the development and usage of split CRISPRa systems in insects have been done before, however we consider this work to be novel and important due our transition of these tools into a vector of disease model. In terms of vector control tools, this will also be the first tool designed to use overexpressing the insects own endogenous genes to produce an effect. It can be used to enhance immune response for example targeting multiple immune genes to increase the effect. Beside putting the basis for building novel control strategies, it can help to study and understand how the immune system responds to virus infection etc…. This is true not only for studying immunity, but for mosquitos for functional studies in general. It can also represent the basis to build stable reproductive barriers that could drive genes through a population in a reversible manner.

We Included more of this information into several red parts throughout the manuscripts to highlight the importance of having this tool now available for vector disease insects.

2) Having said that, the design of the study isn't especially detailed or exploratory of the parameters of the system. Therefore, questions of design of future research will have to be addressed independently of this work - i.e. is this work a future reference for how to best use the CRISPa system in aegypti - probably not. Just that it can work. For example, the work describes two construct designs for expression of sgRNAs, one set has two sgRNA expression cassettes (used for eve and hh) and the second uses two sets of two sgRNAs. The authors state that they do this to increase efficiency - but if this does indeed increase efficiency can’t really be known from their work. While this is in principle understandable - not every design parameter can or should be measurable and/or controlled, this manuscript is packaged as a tool development paper - but how this tool works and can be put together in future work is barely explored.

Another good example of this is the choice in targets: in the first part of the paper they target hh and eve, endogenous transcription factors that regulate the expression of many important developmental genes. As expected, overexpression of either gene is lethal to transgenic individuals, which can be leveraged in applications of engineered genetic incompatibility between two strains (i.e. synthetic species). The authors and other groups have previously developed CRISPa based genetic incompatibility by missexpression of developmental genes in Drosophila, indeed this forms the first half of the incompatibility - the second consists of mutant alleles that retain gene function but are not targetable by dCas9-VPR. Therefore the first part of this manuscript - the encoding of eve/hh based lethality thorough transactivation - does represent a direct step in the process of engineering such genetic control systems.

Unfortunately, the choice of these target genes makes characterizing the CRISPa system in general, difficult since transactivation is expected to lead to dominant lethality. It is also difficult to evaluate off-targeting of a dsCas9-VPR transcription factor when the target genes are themselves transcription factors that in turn activate expression of a number of genes themselves. I think with some improvements to the presentation of the work and sharpening of the narrative this work should be published, hopefully to open further exploration of this tool.

See above comment to point 1. Thank you for this feedback.

Part II – Major Issues: Key Experiments Required for Acceptance

Reviewer #1:

No major issues

Reviewer #2:

1) In Fig.4C is the difference in expression between line A and B here significant? Both lines have similar (mild) efficacy in reducing viral titre or prevalence of infections. Can we assume then that it is pretty insensitive to expression levels of Rel1? Warrants some discussion.

The difference between line A and B is not significant. P Value from one-way ANOVA and Tukey's multiple-comparison test is 0.2244614. We have added this information in the revised manuscript.

2) Four gRNAs were used in the CRISPRa Rel1 set up. Is the effect expected to be cumulative - i.e. more gRNAs more activation?

Yes, it has been shown that CRISPRa applications also benefit from multiplexing and that gene activation is synergistically promoted by multiple dCas9VR/sgRNA binding events at the proximal region of the promoter. In human cells, for example dCas12a-based activators with three gRNAs targeting a single gene increased gene activation by 9- to 40-fold relative to single gRNAs while, with dCas9-VPR, four sgRNAs targeting a single gene enhanced gene activation by more than 1000-fold (Cheng, A. W. et al. Multiplexed activation of endogenous genes by CRISPR-on, an RNA-guided transcriptional activator system. Cell Res. 23, 1163–1171 (2013)). We have added this information in the revised manuscript.

3) In Fig2B were the were crosses 2 and 3 compared against cross 1 to see if significant i.e. is II (VPR only) causing significant overexpression without gRNA? If so, are there overlap between these genes up-regulated and those upregulated in the presence of gRNA?

There is no significant difference between WT, Cas9-VPR and sgRNA control lines: See table below with one-way ANOVA and Tukey's multiple-comparison test (yellow highlighted)

hh transactivation

diff	lwr	upr	p adj

c2-c1	0.362000 -1.521426 2.2454263 0.9242802

transhet-c1 6.596667 4.713240 8.4800930 0.0000168

wt-c1	-0.541750 -2.328525 1.2450251 0.7691721

transhet-c2 6.234667 4.550079 7.9192543 0.0000111

wt-c2	-0.903750 -2.479537 0.6720375 0.3247294

wt-transhet -7.138417 -8.714204 -5.5626292 0.0000024

eve transactivation

diff	lwr	upr	p adj

c2-c1	-1.08166667 -3.202140	1.0388066 0.4138267

transhet-c1 20.64500000 18.524527 22.7654733 0.0000000

wt-c1	-1.09750000 -3.109158	0.9141576 0.3621648

transhet-c2 21.72666667 19.830058 23.6232756 0.0000000

wt-c2	-0.01583333 -1.789949	1.7582819 0.9999909

wt-transhet -21.74250000 -23.516615 -19.9683848 0.0000000

4) The last two concluding statements in the Discussion are so vague I doubt that many will be able to understand how these findings strengthen or enable these aspects

Conclusion statements have been rephrased for clarity.

5) "The extent of viral suppression is stronger than that displayed when using RNAi-mediated gene silencing of the Toll pathway negative regulator Cactus, most likely due to the more robust immune activation of the Toll immune signaling pathway achieved through CRISPRa-mediated transactivation of the Rel1 transcription factor." - is there RNAi data for the targeting of Rel1 transcription factor directly (rather than Cactus)?

RNAi-mediated gene silencing directly targeting Toll pathway positive transcriptional factor, Rel1, resulted in significant induction of the DENV2 viral titers in the mosquito midguts. Further, we have compared the fold-induction of Rel1 expression from RNAi-mediated gene silencing of Cactus with CRISPRa-mediated transactivation of the Rel1 transcription factor. There was 3-fold induction of Rel1 expression through Cactus RNAi gene silencing, compared to an 11-fold induction in the CRISPRa-mediated transactivation system. We have included this information in the revised manuscript.

6) I was interested in the finding that the parental source of the dCas9 or the gRNA made a difference - I suspect this is due to the larger dose of guideRNA that gets into the embryo when inherited maternally rather than any paternal effect associated with delivery of dCAS9. However, none of this was elaborated in the discussion and what it might mean operationally. Also, in later crosses with the Rel1 i assume you do not have the corresponding reciprocal crosses, as above, to see if these difference in parental provision of alleles consistently makes a difference?

We thank the reviewer for the comments. The fact that the lethality effect is higher when dCas9 is inherited paternally and sgRNA maternally lead us to speculate that the high dose of sgRNA through maternal deposition could have allowed to increase the transactivation in a similar way as multiplexing. If the gRNA is the limiting factor to induce an early transactivation, the presence of higher sgRNA dose might have promoted multiple dCas9VR/sgRNA binding events at the proximal region of the promoter and thus increased transactivation. However this is just a speculation and it might be target dependents, so we did not included in the discussion for this reason.

7) "We then searched to see if any nearby gene was upregulated in our RNAseq data. Among 85 potential off-target sites for the eve or hh sgRNAs, only 8 sites correlated to eve sgRNAs, localized near genes that were differentially expressed in our analysis with LogFC > 2 and FDR < 0.05" - syntax and use of (or not) comma are important here. You mean to say 8 out of 85 potential off target sites showed upregulation of nearby genes that would be consistent with off-target binding of the transactivator? Do you have controls here for comparison? if 10% of genes near potential off target sites are differentially regulated, what is the overall percentage of genes differentially regulated? Are all 85 off targets sites near enough to genes to be expected to show differential regulation if they were bound by CRISPRa?

Thank you for this feedback - we have revised this entire section of the text as follows: “To determine if dCas9-VPR has off-target activation effects as described previously [[49,50]](https://paperpile.com/c/TTVGNc/wHW9i+5LcNq), potential off-target binding sites were predicted bioinformatically using an optimal CRISPR target finding software [[51,52]](https://paperpile.com/c/TTVGNc/vfk2Q+dtuYW). We then analyzed each of the predicted off-target binding sites as described in [[45]](https://paperpile.com/c/TTVGNc/XQiVw) and to determine if any closely-linked genes were upregulated in our RNAseq experiments A total of 85 potential off-target sites were detected among all the sgRNA target sites (**Tables S8-9**). The majority of off-target sites reside far from functional loci, in the intronic region, or in the proximity of genes that do not show any differential regulation, based on the RNA sequencing information (**Tables S8-9**). However, eight off-target sites related to *eve* sgRNAs are localized upstream of genes that were differently expressed in our analysis, with significant fold changes higher than two-fold (**Fig. 2C, Tables S8-9**). Not surprisingly, there are some important developmental transcription factors among those genes, including *scabrous* [[53]](https://paperpile.com/c/TTVGNc/LEgs) and *nk* homeobox genes [[54]](https://paperpile.com/c/TTVGNc/Lsw6), indicating that their upregulation is likely mediated by *eve* overexpression rather than off-targeting (**Tables S8-9**). No potential off-target sites related to *hh* sgRNAs showed significant transactivation. These results indicate that the probability of off-target transactivation with dCas9-VPR is low. Nonetheless, the genome-wide differential gene expression is likely either a direct or indirect effect of *eve* and *hh* transactivation, confirming the ability of using this tool to induce an efficient and measurable overexpression.”

Reviewer #3:

1) In results 2: Overexpression of eve and hh generated additional transcriptomic changes. First of all, a comment on the style of narrative. It is written in a difficult to read way, with too many numbers mentioned, and obvious issues glanced over. This reviewer had to read this section multiple times to really understand it, and then discovered what seem to be an important issue - that lead authors likely missed because of how this section is written: 2nd sentence: When reporting on differential expression between transheterozygous and parental strains the authors note that 6488 and 7816 genes are differentially expressed, depending on which of the parental strains are compared - that's around 10% difference - a huge amount. What the (43%) are noted in this sentence is not clear to this reviewer. This difference (and dry reading) continues when another filter is added (logFC > -2|2), although the differences between the two controls becomes smaller. How is it possible that there is such a difference between the controls. This becomes especially relevant perhaps in the ability to detect off-targeting, or sgRNA-independent dCas9-VPR binding and expression. Volcano plots are wholly impractical and insufficient here to describe what is happening, and above half a page of dry information on the number of genes could be summarized in a table or some better plots that properly explore expression including in controls and their appropriate application. The screen-shoting of IGV alignments of the data is amateur, as is the description of the data - for each of the developmental genes targeted, eve and hh, the final sentence of each paragraph describing the RNAseq “analysis” ends with a list of the “most significantly upregulated genes” ordered by ascending significance score. This is based on basic misunderstanding of differential expression - the genes with the lowest significant scores are not the most interesting/significant genes - they are the genes that the algorithm feels most “confident” are differentially distributed in the two datasets. Indeed by looking at the volcano plots, eve and hh are not even themselves the genes with the lowest adjusted p-value.

The whole section has been re-write for clarity and removing jargon and many of the numbers that make the read less fluent.

2) In the same results chapter: the off-targeting analysis is unclear, including because the methods section misses this part of the work. 62 potential off-targets were identified (although how or what is considered an off-target isn't explained) in the genome and the authors then “searched to see if any nearby gene was upregulated”... what is nearby? how are differences quantified? This section needs to be revised and explained appropriately.

The whole section has been re-write for clarity and removing jargon and many of the numbers that make the read less fluent.

3) In the next section describing lethality/toxicity there is no follow-up of the transhets that survive, besides looking at them under the microscope to look for morphological differences. This is not enough. What is the basis for their survival - is this heritable?

We have included some information about the hatched larvae and their expression in the results section: “Moreover, only larvae with *eve* transactivated survive to adulthood, and interestingly those larvae did not show any significant level of upregulation when eve transcript level was tested with qPCR (Fig S3).”

Part III – Minor Issues: Editorial and Data Presentation Modifications

Reviewer #1:

see attached file

Comments addressed above

Reviewer #2:

see attached annotated manuscript for these - it is a lot easier than listing them here. (PDF file)

1) Page 4: “ Furthermore, dCas9-VPR can be directed to nearly any DNA sequence with a sgRNA” : needs to be more precise - I suggest 'by'. As it is currently, reads as though the DNA possesses the sgRNA
2) “However, *in vivo* use of this tool is limited and it has not been demonstrated yet in any disease vectors”
3) “Here we have generated the first CRISPRa system in Ae”: to what purpose? the 'why' has not yet been stated anywhere...
4) Last paragraph of introduction: not really giving the reader a sense of why or how this would be achieved...
5) Page 7: syntax and use of (or not) comma are important here. You mean to say 8 out of 85 potential off target sites showed upregulation of nearby genes that would be consistent with off-target binding of the transactivator? Do you have controls here for comparison? if 10% of genes near potential off target sites are differentially regulated, what is the overall percentage of genes differentially regulated? Are all 85 off targets sites near enough to genes to be expected to show differential regulation if they were bound by CRISPRa?
6) Discussion rephrasing

All comments from the PDF have been addressed

Figures are generally well made, informative and easy to read - a couple of formatting suggestions there

Reviewer #3:

I focus mainly on the results section, given time constraints despite the extent of improvements that are needed

Section 1: Lines

1) Which polyubiquitin gene did you use the promoter of?

We have updated the manuscript with the AAEL# for PUb, AAEL003877.

2) Since you can’t actually prove that having multiple sgRNAs increases efficacy, provide references for where this was shown, or reduce emphasis.

Yes, it has been shown that CRISPRa applications also benefit from multiplexing and that gene activation is synergistically promoted by multiple dCas9VR/sgRNA binding events at the proximal region of the promoter. In human cells, for example dCas12a-based activators with three gRNAs targeting a single gene increased gene activation by 9- to 40-fold relative to single gRNAs while, with dCas9-VPR, four sgRNAs targeting a single gene enhanced gene activation by more than 1000-fold (Cheng, A. W. et al. Multiplexed activation of endogenous genes by CRISPR-on, an RNA-guided transcriptional activator system. Cell Res. 23, 1163–1171 (2013)).

3) Rephrase “two different regions within the promoter region” to “two different sgRNA sites within 250 bp from the TSS”. I initially read this as distinct regions that were selected based on some criteria.

We have changed the phrase to “To increase efficacy, each sgRNA line encodes two different sgRNAs targeting different locations within 250bp upstream of the TSS of the gene of interest” in order to improve clarity.

4) Fig 1 is very pretty, but in Fig1c its very hard to see the eye fluorescent phenotypes.

We have increased the brightness of the zoomed in panels featuring larvae with 3xp3-tdTomato. We have also included arrows pointing to the fluorescent eyes.

Section 2: Overexpression of target genes

5) Explain why 24hr embryos were selected

Because the polyubiquitin promoter is expected to be expressed throughout all time points of *Ae. aegypti* development, we found that any developmental time point could be used to determine if transactivation was present in transheterozygous individuals. We decided to use the 24 hr time point because transcriptomic data showed very low expression of both *eve* and *hh* at that time point. The natural low expression in WT would allow us to potentially detect a greater difference in expression rate if transactivated by the CRISPRa system compared to a time point where either genes were already very active.

6) The final paragraph describing the transcriptomic data is difficult to read and could be much better described in a figure with many of the details going to the methods. The IGV screenshot should be removed. Fold increases of expression are stated but it is not clear in reference to what? I would suggest that a separate bar plot is made depicting TPMs for eve and hh specifically, in addition to dCAs9 and some control for each of the F1 genotypes, similarly to the Fig 1B, showing the qRT-PCR data.

The whole section has been re-write for clarity and removing jargon and many of the numbers that make the read less fluent.

7) It looks to me from looking at the TPM values that there are also big differences in hh expression between parental strains. Given that these genes are developmental, I wonder how the design of the study compromised the clarity of the results and this is hard to understand from the results as they are presented.

There is a slight increase in hh expression in dCas9 parental line (TPM=5) compared to gRNA lines (TPM2), but not at the level of transheterozygotes and mainly due to high variability between samples. There is no difference in hh levels when wt, sgRNA and Cas9 samples were analyzed via qPCR, plus the embryos clearly presented high lethality and showed higher levels of hh via FISH. The purpose of the papers is not to deeply understand the dynamics of *hh* gene function, rather than show efficient transactivation by dCasp-VPR system.

Section 3: additional transcriptomic changes

8) What is 43%? And 41% in eve and hh respectively

The whole section has been re-write for clarity and removing jargon and many of the numbers that make the read less fluent.

9) Fig2B should be Fig2C

We have updated our Figure references.

10) Volcano plots show only one control but the results report important differences

We decided to only show graphs of transheterozygote RNAseq data vs their sgRNA control counterparts to simply demonstrate upregulation of the target genes were occurring as well as the differential expression of numerous other genes. However, additional controls performed with our RNAseq analysis can be found in TableS2, S5, and S6.

11) Off-target analysis is unclear (see above)

The whole section has been re-write for clarity and removing jargon and many of the numbers that make the read less fluent.

12) Reporting on the top genes based on significance is not appropriate. Instead the authors should at least attempt to categorize these genes based on whether they are known or not as being downstream targets of eve or hh

They are categorized based on fold changes not only P value.

Eve- repressor for engrailed (indirectly through paired, runt, and sloppy paired), fushi tarazu, ultrabithorax, and wingless

Hh - induces decapentaplegic and wingless

13) Off-targets that are within 250 bp of TSS should be relevant only in theory, but i dont see how the position of the off-target in relation to the candidate gene is linked.

The whole section has been re-write for clarity and removing jargon and many of the numbers that make the read less fluent.

14) The sentence “ Furthermore, the probability of off-target transactivation with dCas9-VPR is likely low” should be referenced, or deleted.

The whole section has been re-write for clarity and removing jargon and many of the numbers that make the read less fluent.

Section 4: Lethality

15) Fig3A does not show what is mentioned in the second sentence.

We have updated our Figure references

16) I have issues with Fig3A in general - pictures of larvae to say that nothing was observed is not ok.

We felt the need to show images of transheterozygous larvae where “nothing was observed” because it we wanted to show that there were individuals that escaped the effects of CRISPRa transactivation and their phenotypes were contrary to expectations.

17) Rephrase: “targeting both genes…”. This can mislead readers to think that a cross between the two sgRNAs, to make individuals containing both were made.

We have rephrased the sentence to make it clear that we are only targeting one gene at a time.

18) Again difficult to read what the results are, because they are buried in details in whether the cas9-vpr was paternal or maternal in origin. Given this, results are mentioned but their significance cannot be extracted easily.

The whole section has been re-write for clarity and removing jargon and many of the numbers that make the read less fluent.

19) The in situs that were done to explain the lethality are pretty unimpressive and lacking controls. Sure I will take your word for it. Why were in situs done at this late stage of embryonic development, when the pair rule gene pattern is no longer clearly visible.

We tried earlier time points but they were too fragile for good fixation and dissection. 24hrs allowed for good samples while also seeing active overexpression of hh. We also were able to notice a more drastic developmental difference between 24 hr WT and 24 hr tranhets with the latter being less developed within the same period.

Section 5: Rel1 overexpression:

20) Why did authors choose to increase yet again the number of sgRNAs?

It has been shown that CRISPRa applications also benefit from multiplexing and that gene activation is synergistically promoted by multiple dCas9VR/sgRNA binding events at the proximal region of the promoter. In human cells, for example dCas12a-based activators with three gRNAs targeting a single gene increased gene activation by 9- to 40-fold relative to single gRNAs while, with dCas9-VPR, four sgRNAs targeting a single gene enhanced gene activation by more than 1000-fold (Cheng, A. W. et al. Multiplexed activation of endogenous genes by CRISPR-on, an RNA-guided transcriptional activator system. Cell Res. 23, 1163–1171 (2013)).

21) Why is the difference between the transhets and the controls, in terms of median viral titers described twice, once as a 3-fold reduction and once as a 18% reduction. I am confused on the difference between these sentences.

3-fold reduction indicates the viral titers, while 18% reduction indicates the infection prevalence. Both infection intensities (viral loads) and infection prevalence (% of infected mosquitoes with at least one virion) are both key factors to determine the significance of infection differences.

22) Please rephrase the conclusion of your result here from : consistent with previously published conclusion” to what that conclusion actually was.

Conclusion statements have been rephrased for clarity.

23) It is really a shame that no followup of this was mentioned - feels like the minimum published result of regulating gene expression for immunity engineering.

We agree with the reviewer’s comments. Here we have included more information and discussed further the regulation of gene expression by including results we have obtained from the CRISPRa-mediated transactivation of Rel2, the IMD immune pathway transcriptional regulator. We have also discussed the disadvantages of using current CRISPRa-mediated regulations of the immune genes when the expression of dCas9-VPR was driven by a polyubiquitin (PUb) promoter known to be active at relatively high expression levels within a variety of cell types and developmental stages. The regulations of immune genes require fine-tuning, and the spatiotemporal activations of the immune genes are desirable. The development of the tissue-specific expression of dCas9-VPR is undergoing to address these concerns.

Other instances:

24) Reference 71 is repeated several times, and I believe some additional important references need to be added here from other labs working on the same system, not just the outputs of this group.

References fixed
